# Prevalence of general and abdominal obesity in Portugal: comprehensive results from the National Food, nutrition and physical activity survey 2015–2016

**DOI:** 10.1186/s12889-018-5480-z

**Published:** 2018-05-11

**Authors:** Andreia Oliveira, Joana Araújo, Milton Severo, Daniela Correia, Elisabete Ramos, Duarte Torres, Carla Lopes, by the IAN-AF Consortium, Carla Lopes, Andreia Oliveira, Milton Severo, Duarte Torres, Sara Rodrigues, Elisabete Ramos, Sofia Vilela, Sofia Guiomar, Luísa Oliveira, Violeta Alarcão, Paulo Nicola, Jorge Mota, Pedro Teixeira, Simão Soares, Lene Frost Andersen

**Affiliations:** 1 EPIUnit – Institute of Public Health, University of Porto, Rua das Taipas, 135-139 4050-600 Porto, Portugal; 2 Department of Public Health and Forensic Sciences, and Medical Education, Unit of Epidemiology, Faculty of Medicine, University of Porto, Porto, Portugal; 3 Faculty of Food and Nutrition Sciences, University of Porto, Porto, Portugal

**Keywords:** National Survey, Obesity, Overweight, Prevalence

## Abstract

**Background:**

This study includes, for the first time, estimates of general and abdominal obesity prevalence for all ages of the Portuguese population, using common standardized methodologies. Results are compared by sex, age groups, educational level and geographical regions.

**Methods:**

Participants were a representative sample of the Portuguese population aged between 3 months and 84 years of age (*n* = 6553), enrolled in the National Food, Nutrition and Physical Activity Survey, 2015–2016. Objective anthropometric measurements included length/height, weight and body circumferences, performed according to standard procedures. Body mass index (BMI) was classified according to the World Health Organization (WHO) growth charts for children and adolescents, and WHO criteria for adults. Abdominal obesity was defined in adults as waist-hip ratio ≥ 0.85 in women or ≥ 0.90 in men. Prevalence estimates and 95% confidence intervals (95%CI) were weighted according to a complex sampling design, considering stratification by seven geographical regions and cluster effect for the selected Primary Health Care Unit.

**Results:**

The national prevalence of obesity is 22.3% (95%CI: 20.5–24.0), significantly higher in women. Obesity prevalence is much higher in the elderly (39.2%, 95%CI. 34.2–44.2), while children and adolescents have the lowest prevalence around 8–9%. In a regression model, three knot points denoting an inflection of obesity prevalence across the life span were observed around 5, 15 and 75 years.

The prevalence of pre-obesity at national level is 34.8% (95%CI: 32.9–36.7), higher in men, and almost 18% of children and 24% of adolescents have pre-obesity.

The sex- and age-standardized prevalence of obesity ranged from 38.3% (95%CI: 34.6–42.1) to 13.1% (95%CI: 10.3–15.9) for the less and the most educated individuals, respectively. Although some geographical region disparities, obesity prevalence did not significantly differed across regions (*p* = 0.094).

The national prevalence of abdominal obesity in adults is 50.5% (95%CI: 47.9–53.1), particularly high in the elderly (80.2%).

**Conclusion:**

Almost 60% of the general Portuguese population is obese or pre-obese. Women, elderly and less educated individuals present the highest obesity prevalence. Abdominal obesity, in particular, seems to be a relevant public health problem among the elderly men.

**Electronic supplementary material:**

The online version of this article (10.1186/s12889-018-5480-z) contains supplementary material, which is available to authorized users.

## Background

Obesity has well-known associated health consequences, with long-lasting effects on morbidity, such as increased risk of diabetes, cardiovascular diseases, and cancer, but also has established effects on premature mortality [1]. It has a high burden at the population level [2, 3] also because of its epidemiological distribution; the prevalence of overweight and obesity has increased substantially over the past three decades. Recent estimates point out an epidemiological transition from underweight to overweight and obesity throughout the world [4]. Globally, the proportion of adults with a body mass index (BMI) of 25.0 or greater increased from 28.8% in 1980 to 36.9% in 2013 for men and from 29.8% to 38.0% for women, in both developed and developing countries. There have also been substantial increases in the prevalence among children and adolescents, with 23.8% of boys and 22.6% of girls in developed countries being either overweight or obese in 2013 [5].

Concerns about the health and economic burden of increasing BMI have led to the inclusion of adiposity among the global non-communicable disease targets [3]. The need for preventive actions of obesity is increasingly acknowledged, but the World Health Organization (WHO) highlights that actions need to be systematic, evidence-based and stakeholder-informed [6]. In fact, there have been widespread calls for regular monitoring of changes in overweight and obesity prevalence in all populations [7].

In Portugal, data on obesity prevalence based on measured weight and height is only available for some age groups and some of them are out of date. In 2016, the National Health Examination Survey with Physical Exam (INSEF) has provided the prevalence of measured obesity, but only adults 25 to 74 years were included in these estimates [8]. Prior estimates in adults have been restricted to self-reported data from national health surveys that provided useful trends over time [9], or objectively measured data from national surveys conducted some years ago [10, 11]. For pediatric age, available data include nationwide estimates from the WHO European Childhood Obesity Surveillance Initiative (COSI), but only for 6- to 9-year-old children [12, 13]. Obesity prevalence for Portuguese adolescents is available at the national level from the Health Behaviour in School-aged Children (HBSC), a WHO cross-national survey [14, 15], but estimates are based on self-reported weight and height. Obesity prevalence in adolescents based on objective measures is available at the national level from 2008 [16] or from other studies only at regional or community level, and for specific age ranges.

The National Food, Nutrition and Physical Activity Survey was conducted in 2015–2016, and includes objective measurements of weight, height, waist and hip circumferences for a broad age range of the population (from 3 months to 84 years), using standardized objective measurements. This Survey provides updated nationwide and regional data on obesity, able to assist Portugal with official indicators at the European level. Therefore, this study aims to describe the distribution of general and abdominal obesity in the Portuguese population, by sex, age groups, educational level and geographical regions.

## Methods

Participants were enrolled in the National Food, Nutrition and Physical Activity Survey, 2015–2016 (Portuguese acronym: IAN-AF 2015–2016 Survey), which aimed to collect nationwide and regional data on dietary habits, physical activity and anthropometrics, and to evaluate their relation with other determinants, such as socioeconomic factors. The IAN-AF 2015–2016 Survey was conducted by a Consortium, involving researchers from the University of Porto (Promoter), the University of Lisbon, the National Institute of Health (INSA), the University of Oslo, Norway and the enterprise SilicoLife.

A representative sample of the Portuguese general population, aged between 3 months and 84 years of age, was selected from the National Health Registry, by multistage sampling, in each of the seven Portuguese geographical regions (NUTSII) and weighed according to sex and age groups. Individuals living in collective residences or institutions, living in Portugal for less than 1 year (non-applicable to infants), non-Portuguese speakers, with diminished physical and/or cognitive abilities that hamper participation or dead were excluded.

Two interviews (8 to 15 days apart) were conducted by trained researchers with background in Nutrition or Dietetics, by using Computer-assisted personal interviewing (CAPI), during 12 months (from October 2015 to September 2016). The examination site was the participant’s home (< 1%) or the Primary Health Care Unit they belong to, selected according to participant’s preference.

A total of 6553 individuals completed the first interview (during which anthropometrics were assessed). The participation rate (calculated as participants divided by eligible plus unknown eligible individuals) was 26.0%. The cooperation rate (participants/eligible individuals) was 33.4%.

An electronic platform (You eAT & Move) was developed to manage the field work and to assist data collection. This e-platform includes the You' module to collect sociodemographic and other health-related data, namely to register anthropometric measurements; the ‘eAT24’ module for collection of food consumption data by a 24-h recall (or food diaries); and the ‘MOVE’ module for data collection on physical activity. Most of the procedures of data collection were adapted from the European Food Safety Authority Guidance in view of the EU Menu methodology [17].

More detailed information on design and methods of the IAN-AF 2015–2016 Survey is described elsewhere [18].

### Anthropometrics

Objective anthropometric measurements, including length/height, weight and body circumferences, were performed in both children and adults according to standard procedures [19], by trained observers. Data on anthropometrics retrieved from the health booklets or self-reported were also assessed, but in the current study were only used to check validity of the objective measurements.

Height was measured to the nearest centimetre, with participants in a stand position with light clothing and barefoot, using a portable wall stadiometer (SECA® 213, Hamburg, Germany). For children with less than 2 years of age, recumbent length was measured to the nearest 0.1 cm with a measuring rod with large callipers (SECA® 207; Hamburg, Germany).

Body weight was measured in the same conditions, to the nearest tenth of a kilogram using a digital scale (SECA® 813, Hamburg, Germany). For children with less than 2 years of age, a specific pediatric digital weight scale was used (SECA® 354, Hamburg, Germany) and measurements were performed with participants naked and, whenever accepted, without diaper, to the nearest 0.01 cm.

Waist and hip circumferences were measured in all age groups except in children of less than 3 years of age and in pregnant women. Waist circumference was measured at the level of the narrowest point between the lower costal border and the top of the iliac crest, perpendicular to the long axis of the trunk. The measurement was taken at the end of a normal expiration. Hip circumference was measured at the level of greatest posterior protuberance of the buttocks, perpendicular to the long axis of the trunk. All these body circumferences were measured on the skin using an anthropometric tape, to the nearest 0.1 cm, with the subject in a relaxed standing position, with the feet slightly apart and mass equally distributed on both feet.

More detailed information on the measurement procedures and conditions could be found in the Procedures Manual of the project, available in Portuguese through the website ian-af.up.pt.

Some quality control procedures were adopted: initial training with a certificated anthropometrist and on-going training by Regional Coordinators of field work, in some cases by using distance electronic devices; a bubble level was used to check the best position for the equipment in the room; a small platform was used to allow the direct observation of values from the stadiometer; the calibration of scales using standard weights of 5000 g and 500 g and their combinations was performed; to check possible information bias, preliminary statistical analysis during fieldwork was conducted, namely comparing the distribution of participant’s anthropometrics by interviewer; and related doubts were registered in an editor book to be solved by the research team.

Body mass index was calculated as weight over the squared height and three main categories - underweight/normal weight, pre-obesity and obesity - were defined according to the WHO standards (for children and adolescents age and sex-specific BMI z-scores were used) [20–23]. Out of the 6553 individuals who performed the first interview, 6235 had weight and length/height measured and valid. Pregnant women (*n* = 59), a subject with dwarfism and eight individuals in critical conditions, namely with oedema and tubes, were excluded from analysis, totalling 6167 individuals (3208 women and 2959 men).

In adults (≥18 years), abdominal obesity was also defined according to waist-hip ratio, available for 4012 individuals. A substantially increased risk of metabolic complications was defined if the waist-hip ratio was ≥0.85 in women or ≥ 0.90 in men, according to the WHO criteria [24].

### Socioeconomic data

Sex and age were automatically imported from datasets obtained from the National Health Registries. Age was calculated by the subtraction between the evaluation date and birth date. These data were checked during the first contact with the participants.

The number of completed years of schooling was asked to each participant. For children and adolescents, the highest number of completed years of schooling of one of the parents was considered. Three categories of educational level were defined (no formal education to 2nd cycle of basic education, 3rd cycle of basic education to high school, and higher education).

The geographical region of each participant was decided based on the location of the Primary Health Care Unit to which participants belong to. Seven Statistical Geographical Units - NUTS II (North, Centre, Lisbon Metropolitan Area, Alentejo, Algarve, Madeira and Azores) were considered.

### Statistics

Prevalence estimates were weighted according to the complex sampling design, considering stratification by the seven Portuguese geographical regions (NUTS II) and cluster effect for the selected Primary Health Care Unit. The respective 95% confidence intervals (95%CI) were provided.

Prevalence estimates according to sex, age groups, educational level or geographical region were compared by the Qui-squared test. When comparing estimates by geographical regions or by educational levels, standardization for sex and age considering the distribution of the Portuguese population, according to data from the last CENSUS [25], was also performed.

To evaluate the trend of obesity prevalence with age, B-Spline basis for polynomial splines generalized additive models within weighted logistic regression were used [26].

A significance level of 5% was assumed. Analyses were performed using the library “survey” of R software (The R Project for Statistical Computing), version 3.4.0 for Windows.

## Results

The national prevalence of obesity is 22.3% (95%CI: 20.5–24.0), significantly higher in women (24.3%, 95%CI: 21.9–26.7 vs. 20.1%, 95%CI: 17.5–22.7). Obesity prevalence increases with increasing age, with the lowest prevalence in children (7.7%, 95%CI: 4.6–10.9) and the highest in the elderly (39.2%, 95%CI: 34.2–44.2) (Table 1). In each age group, no significant differences were found between sexes (Additional file 1: Table S1).

**Table 1:** Prevalence of body mass index categories^a^, for the national sample, by sex and age groups

	National	Women	Men	Children (< 10 years)	Adolescents (10–17 years)	Adults (18–64 years)	Elderly (65–84 years)
Obesity							
n	1198	674	524	73	72	777	276
$\widehat{N}$	2.244.500	1.232.890	1.011.610	59.282	74.406	1.488.445	622.367
weighted % ^b^	22.3%	24.3%	20.1%	7.7%	8.7%	21.6%	39.2%
95% CI	[20.5–24.0]	[21.9–26.7]	[17.5–22.7]	[4.6–10.9]	[5.5–12.0]	[19.5–23.8]	[34.2–44.2]
Pre-obesity							
n	1830	830	1000	173	163	1181	313
$\widehat{N}$	3.506.169	1.553.537	1.952.632	132.074	200.559	2.509.942	663.594
weighted % ^b^	34.8%	30.7%	38.9%	17.3%	23.6%	36.5%	41.8%
95% CI	[32.9–36.7]	[28.1–33.2]	[36.0–41.7]	[13.7–20.8]	[19.6–27.5]	[34.2–38.8]	[36.5–47.0]
Underweight/normal weight							
n	3139	1704	1435	1217	457	1323	142
$\widehat{N}$	4.334.756	2.277.584	2.057.173	573.687	576.374	2.882.384	302.312
weighted % ^b^	43.0%	45.0%	41.0%	75.0%	67.7%	41.9%	19.0%
95% CI	[40.7–45.2]	[42.0–48.0]	[37.9–44.1]	[70.8–79.1]	[63.3–72.1]	[39.2–44.5]	[14.1–24.0]
*p*-value ^c^		*p* = 0.024		*p* < 0.001			

*n* sample size, $\widehat{N}$ estimated population size, *95%CI* 95% confidence intervals

^a^BMI categories defined according to the World Health Organization criteria

^b^Prevalence weighted for the distribution of the Portuguese population

^c^*P*-value comparing the prevalence of obesity vs. the other categories

An additional analysis was conducted, estimating the obesity prevalence by a regression model, based on the observed prevalence by 5-years age groups (Additional file 2: Figure S1). Three knot points denoting an inflection of obesity prevalence across the life span were observed around 5, 15 and 75 years.

The prevalence of pre-obesity at national level is 34.8% (95%CI: 32.9–36.7). Pre-obesity is higher in men (38.9%, 95%CI: 36.0–41.7), than in women (30.7, 95%CI: 28.1–33.2), and in the elderly (41.8%) (Table 1). Almost 18% of children and 24% of adolescents have pre-obesity. Due to the very low prevalence of underweight individuals (1%), this category was merged with normal weight group. Approximately 40% of the Portuguese population (43.0%, 95%CI: 40.7–45.2) is underweight/normal weight.

The prevalence of obesity and pre-obesity is significantly higher in the less educated individuals. The national sex and age-standardized prevalence of obesity ranged between 38.3% (95%CI: 34.6–42.1) and 13.1% (95%CI: 10.3–15.9) for the less and the most educated individuals, respectively. Prevalence differences according to the educational level were less evident in children and adolescents than in adults (≥18 years) and among these, much more evident for obesity prevalence (Table 2).

**Table 2:** Prevalence of body mass index categories^a^, by educational level^b^ and age groups

	National			< 18 years			≥18 years		
	None,1st and 2nd cycle	3rd cycle and high school	Higher education	None,1st and 2nd cycle	3rd cycle and high school	Higher education	None,1st and 2nd cycle	3rd cycle and high school	Higher education
Obesity									
n	592	435	169	26	85	34	566	350	135
$\widehat{N}$	1.115.485	797.234	325.902	27.695	77.245	28.749	1.087.790	719.989	297.153
weighted %^c^ (95%CI)	38.5 [34.8–42.2]	17.1 [14.8–19.3]	13.2 [10.3–16.1]	13.0 [7.3–18.6]	9.9 [7.1–12.7]	4.8 [2.2–7.3]	40.6 [36.7–44.4]	18.5 [15.9–21.1]	15.9 [12.2–19.6]
standardized %^d^ (95%CI)	38.3 [34.6–42.1]	16.9 [14.8–19.1]	13.1 [10.3–15.9]	13.0 [7.2–18.7]	9.8 [7.3–12.2]	4.6 [2.3–6.9]	40.4 [36.5–44.3]	18.4 [15.9–20.9]	15.8 [12.3–19.3]
Pre-obesity									
n	611	786	426	53	161	118	558	625	308
$\widehat{N}$	1.124.068	1.612.171	759.117	55.865	152.021	120.569	1.068.204	1.460.150	638.548
weighted %^c^ (95%CI)	38.8 [34.8–42.8]	34.5 [31.8–37.2]	30.7 [27.6–33.8]	26.2 [18–34.3]	19.5 [15.5–23.6]	20.0 [16.0–23.9]	39.8 [35.6–44.1]	37.5 [34.4–40.6]	34.2 [30.2–38.3]
standardized %^d^ (95%CI)	39.2 [35.2–43.2]	34.7 [32.1–37.4]	30.9 [27.7–34.0]	26 [18.0–34.0]	19.1 [15.3–23.0]	20.4 [16.4–24.4]	40.3 [36.0–44.6]	37.9 [34.9–41.0]	34.2 [30.1–38.2]
Underweight/normal weight									
n	436	1570	1119	159	822	680	277	748	439
$\widehat{N}$	656.053	2.262.101	1.385.222	130.035	548.491	455.011	526.018	1.713.609	930.211
weighted %^c^ (95%CI)	22.7 [19.2–26.1]	48.4 [45.3–51.5]	56.1 [52–60.2]	60.9 [52.1–69.7]	70.5 [66.1–75.0]	75.3 [70.9–79.6]	19.6 [16.1–23.1]	44 [40.5–47.5]	49.9 [44.8–54.9]
standardized %^d^ (95%CI)	22.5 [19.2–25.8]	48.3 [45.4–51.2]	56 [51.9–60.0]	61.0 [52.3–69.7]	71.1 [66.9–75.4]	75.0 [70.8–79.3]	19.3 [15.9–22.6]	43.7 [40.3–47.0]	50.0 [45.0–55.0]
p-value^e^	*p* < 0.001			*p* = 0.004			p < 0.001		

*n* sample size, $\widehat{N}$ estimated population size, *95%CI* 95% confidence intervals

^a^BMI categories defined according to the World Health Organization criteria

^b^Subjects with missing value in education were excluded from analysis (*n* = 23)

^c^Prevalence weighted for the distribution of the Portuguese population

^d^Prevalence standardized for sex and age

^e^*P*-value comparing the prevalence of obesity vs. the other categories

**Fig. 1:**
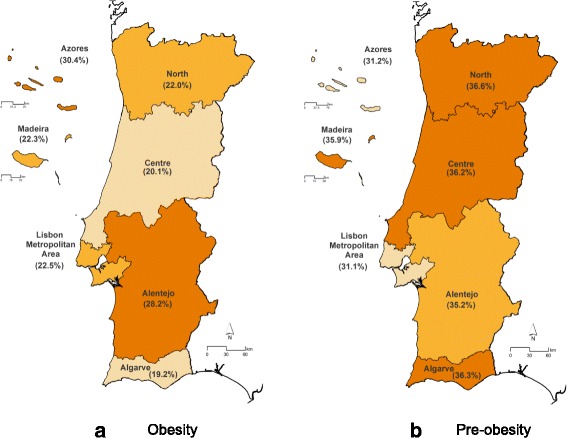
Spatial distribution of the national prevalence of obesity (**a**) and pre-obesity (**b**)* (by the seven Portuguese geographical regions – NUTS II), weighted for the distribution of the Portuguese population. *Obesity categories were defined according to the World Health Organization criteria. Prevalence estimates were mapped using the software ArcGIS® version 10.4, specifically for the IAN-AF Consortium use

Figure 1 shows the spatial distribution of obesity and pre-obesity prevalence in the national sample, according to the seven geographical regions. For comparing prevalence across regions, we standardized estimates for sex and age. Standardized obesity prevalence is higher in Azores (32.9%) and Alentejo (27.6%), and lower in the Center (19.0%) and North (21.5%) of the country (results only shown in text), close to the national prevalence (22.3%). For pre-obesity, the estimates were higher in Madeira (37.0%) and Algarve (37.3%). Although some geographical region disparities, differences between obesity prevalence across regions were not statistically significant (*p* = 0.094).

The national prevalence of abdominal obesity (defined based on a waist-hip ratio of substantially increased risk of metabolic complications) in adults is 50.5% (95%CI: 47.9–53.1), significantly higher in men (62.0% vs. 39.2%) and much higher in the elderly (80.2% vs. 42.7% in adults < 65 years) (Table 3). Lower educational level was also associated with higher prevalence of abdominal obesity, after standardization for age and sex, although in the elderly the disparities due to the educational level are smaller. Age- and sex-standardized prevalence of abdominal obesity is higher in Azores (61.7%) and in the Center region (59.8%), and lower in the Lisbon Metropolitan Area (45.5%) and in the North (47.9%) (Table 3).

**Table 3:** Prevalence of substantially increased risk of waist-hip ratio^a^ in the national sample of adults (≥18 years), by sex, age group, geographical region and educational level

	n	$\widehat{N}$	weighted % ^b^	95% CI	standardized % ^c^	95% CI
National (≥18 years)						
Total	4012	4.424.680	50.5%	[47.9–53.1]	–	–
Women	2120	1.729.600	39.2%	[35.8–42.5]		
Men	1892	2.695.080	62.0%	[58.9–65.1]		
Adults (18–64 years)						
Total	3281	2.959.422	42.7%	[40.1–45.2]	–	–
Women	1766	1.008.970	29.5%	[26.6–32.5]		
Men	1515	1.950.453	55.4%	[51.9–58.8]		
Elderly (65–84 years)						
Total	731	1.465.258	80.2%	[75.7–84.6]	–	–
Women	354	720.630	71.9%	[65.0–78.9]		
Men	377	744.628	90.1%	[85.1–95.2]		
Educational level						
National						
None,1st and 2nd cycle	1401	2.209.083	76.0%	[72.7–79.3]	76.1%	[72.9–79.3]
3rd cycle and high school	1723	1.579.598	40.1%	[36.7–43.5]	40.4%	[37.0–43.8]
Higher education	882	611.000	32.2%	[26.6–37.9]	32.7%	[27.7–37.7]
Adults (18–64 years)						
None. 1st and 2nd cycle	857	1.158.302	71.0%	[66.5–75.5]	70.9%	[66.5–75.3]
3rd cycle and high school	1597	1.281.906	35.9%	[32.4–39.3]	36.1%	[32.6–39.5]
Higher education	826	519.214	30.0%	[25.1–34.9]	30.5%	[26.2–34.7]
Elderly (65–84 years)						
None. 1st and 2nd cycle	544	1.050.781	82.4%	[77.1–87.6]	82.7%	[77.6–87.8]
3rd cycle and high school	126	297.692	82.1%	[74.1–90.1]	82.6%	[74.9–90.3]
Higher education	56	91.786	55.7%	[35.1–76.3]	55.2%	[33.6–76.7]
Geographical region						
North	697	1.441.260	47.4%	[42.4–52.5]	47.9%	[42.7–53.2]
Centre	657	1.219.171	61.4%	[55.2–67.6]	59.8%	[54.1–65.5]
Lisbon Metropolitan Area	526	1.022.659	44.1%	[39.6–48.6]	45.5%	[41.3–49.7]
Alentejo	483	309.731	52.3%	[47.3–57.3]	50.1%	[46.5–53.7]
Algarve	494	190.055	50.5%	[46.9–54.1]	50.6%	[47.5–53.8]
Madeira	566	113.373	48.6%	[42.2–55.0]	51.5%	[46.3–56.6]
Azores	589	128.431	58.6%	[51.5–65.7]	61.7%	[54.6–68.8]

Comparisons of abdominal obesity prevalence between sexes, educational level and geographic region were all statistically significant (p < 0.001; only when comparing educational level in the elderly p = 0.004)

*n* sample size, $\widehat{N}$estimated population size, *95%CI* 95% confidence intervals

^a^Category of substantially increased risk of metabolic complications defined according to the World Health Organization criteria (waist-hip ratio ≥ 0.85 in women and ≥ 0.90 in men) [34]

^b^Prevalence weighted for the distribution of the Portuguese population

^c^Prevalence standardized for sex and age

## Discussion

More than 20% of the Portuguese population is obese, according to the WHO criteria, and six in each ten Portuguese are overweight (pre-obese or obese). Obesity prevalence is higher in women (24.3% vs. 20.1%), and among the elderly (39.2%).

The magnitude of obesity is massive around the world. According to a WHO report, in 2014, 11% of men and 15% of women aged 18 years and older were obese worldwide [3]. Our estimate for Portugal is even higher that the worldwide prevalence (22% of adults and 39% of the elderly are obese), putting into evidence the importance and priority of this public health challenge in our country. In all WHO regions, women are more likely to be obese than men, corroborating our results, that could be further explained by factors related with the hormonal milieu and the environmental and behavioural determinants which predisposes women to excessive weight gain across the life span [27].

The increased obesity prevalence among the elderly is of outstanding note. A more detailed analysis, estimating obesity prevalence in a regression model denoted a peak of obesity prevalence around 70–75 years, decreasing thereafter (data provided in Additional file 2: Figure S1). This knot point at 75 years (representing an inflection of the obesity prevalence) could be explained by a higher survival of those with less accumulation of fat and less health-metabolic complications. We cannot also rule out the fact that our sample is a representative sample of the general population, not including institutionalised elderly (with higher odds of undernutrition), which may lead to an overestimation of obesity prevalence in this age group.

In Portugal, data obtained at the national level on a regular basis is limited to the National Health Surveys, based on self-reported weight and height. The last National Health Survey 2014 [28] reported 16.4% of obesity among individuals of 18 years or older, which is likely to be underestimated due to the self-reported nature of measurements. The First National Examination Survey with Physical Exam (INSEF 2015) [8] has provided the prevalence of obesity and pre-obesity based on measured BMI, and according to WHO criteria, methodologies comparable with our Survey. However, the sampling frame was not the same and they have only included adults 25 to 74 years of age, for which a national prevalence of 28.7% was reported (32.1% in women and 24.9% in men). The obesity prevalence by age groups was also similar, increasing with age categories.

Other national estimates performed in the past for the adult population (+ 18 years) [10, 11, 29] have reported lower obesity prevalence, which could reflect different samplings and methodologies, or an expected increase of obesity prevalence over time, corroborated with worldwide trends [4, 5].

Half of the adult population has abdominal obesity, meaning a substantially increased risk of metabolic complications. The prevalence is higher in males and increases with age, reaching 90.1% in males of 65 years or older. These results are in accordance with data from INSEF [30], and reinforce the need for community interventions, targeting in particular the elderly.

In the present study, abdominal obesity was defined based on waist-hip ratio, a measure that takes into account the effect of abdominal fat but also of peripheral fat (located in the upper and lower members). There is evidence that peripheral fat may have a protective effect on cardiovascular outcomes [31, 32]. Thus, waist-hip ratio seems to reflect the separate and opposing metabolic effects of central and peripheral adiposity, which supports its use as a measure of central adiposity with high cardiometabolic impact.

When considering only the waist circumference estimate (highly correlated with BMI), prevalence estimates are in general lower; the national prevalence in adults is 34.2% (95 CI%: 31.8–36.6), higher in women (41.6%, 95% CI: 38.3–44.9 vs. 26.7%, 95% CI: 23.6–29.8), and much higher in the elderly (≥65 years: 62.4%, 95% CI: 57.7–67.1). Other measures, such as waist-to-height have been described as an indicator of abdominal obesity [33, 34], however a recent studied have highlighted that when compared with DXA measurements, waist-height ratio was more strongly correlated with total fat than abdominal fat in 7 years-old children [35].

In our Survey, estimates for paediatric age showed that 7.7% of children and 8.7% of adolescents are obese and 17.3 and 23.6%, respectively, are pre-obese. The prevalence estimates in children are lower in comparison to the results from COSI – a surveillance system which collects comparable data across Europe each 2–3 years in school-aged children, from 6 to 8 years. The second round of the study conducted in 2009–2010 showed an obesity prevalence of 12.2 and 14.2%, in girls and boys, and for pre-obesity 24.0 and 17.3%, respectively [13]. These differences may be explained by the different age ranges, since the COSI have only evaluated school-aged children. Moreover, in their last report with results from the last round (2015–2016) [36], it was highlighted that the prevalence of childhood obesity is declining in Portugal. It was found a decline of 7.2% for pre-obesity and a decline of almost 4% for obesity between 2008 and 2016, which is a promising result.

Other national survey also conducted in 2009–2010 among 3 to 10-year-old children reported 8.2% of obesity and 19.7% of pre-obesity [37], but the classification was performed according to the International Obesity Task Force cut-offs, which hampers direct comparisons with our estimates based on the WHO criteria.

Among adolescents, the most updated estimates based on national surveys were based on self-reported weight and height from the HBSC/WHO 2014 survey [15], and therefore may be underestimated. The most recent national study collecting objective data in adolescents was performed in 2008 and showed lower prevalence of pre-obesity and obesity in boys, in comparison to our estimates, but higher estimates in girls [16]. However, we are not able to explain if these differences reflect actual temporal trends in obesity prevalence or methodological differences between the studies, namely on sampling procedures and standardization of the estimates. Therefore, challenges in conducting national surveys should be deeply discussed, in order to overcome major limitations, such as the harmonization of methodologies and the relatively low participation rates, often described in population-based national surveys, such as the current one.

A report from WHO [6] has set tools for Member States to determine and identify priority areas for action in the field of population-based prevention of childhood obesity. In Portugal, some programs have been established under the public action of the General Directorate of Health, promoting healthy lifestyles, namely eating habits and physical activity at early ages. Nonetheless, a more systematic and comprehensive approach, at the national level, should be define to tackle childhood obesity.

## Conclusions

Findings from this study provide national and regional updated knowledge on the distribution of measured anthropometrics according to sex, age, education and geographical region. It includes, for the first time, estimates of obesity prevalence for all age ranges of the population, using common standardized methodologies. It will serve as an important descriptive starting point for future follow-up surveys in specific target groups, and will assist public health officials with the information needed to provide official indicators at the European level. Public efforts should be done in order to assure a systematic surveillance system on obesity in Portugal, with comparable methodologies for different age groups.

## Additional files

Additional file 1: **Table S1.** Prevalence of body mass index categories*, by age groups and sex. (DOCX 17 kb)

Additional file 2: **Figure S1.** Prevalence of obesity observed and estimated by a regression model* from 3 months to 84 years. (DOCX 18 kb)

## Abbreviations

BMI: Body mass index
CAPI: Computer-assisted personal interviewing
COSI: European Childhood Obesity Surveillance Initiative
HBSC: Health Behaviour in School-aged Children
IAN-AF 2015–2016 Survey: National Food, Nutrition and Physical Activity Survey, 2015–2016
INSEF: National Health Examination Survey with Physical Exam
WHO: World Health Organization
