# Supplementary material for: Prevalence of general and abdominal obesity in Portugal: comprehensive results from the National Food, nutrition and physical activity survey 2015–2016
Source: BMC Public Health. 2018 May 11;18:614. doi: 10.1186/s12889-018-5480-z (PMC5946450; doi:10.1186/s12889-018-5480-z)
Supplement: Supplementary file 1 — Table S1. Prevalence of body mass index categories*, by age groups and sex. (DOCX 17 kb) [file 12889_2018_5480_MOESM1_ESM.docx]

Supplemental Table 1. Prevalence of body mass index categories^a^, by age groups and sex

|  |  | Children (<10 years) | | Adolescents (10-17 years) | | Adults (18-64 years) | | Elderly (65-84 years) | |
| --- | --- | --- | --- | --- | --- | --- | --- | --- | --- |
|  |  | Women | Men | Women | Men | Women | Men | Women | Men |
| Obesity | | | | | | | | | |
| n | | 36 | 37 | 29 | 43 | 447 | 330 | 162 | 114 |
| $\hat{N}$ | | 25.817 | 33.465 | 24.319 | 50.087 | 804.222 | 684.222 | 378.532 | 243.835 |
| weighted % ^b^ | | 6.9% | 8.5% | 5.9% | 11.4% | 23.7% | 19.7% | 43.0% | 34.5% |
| 95% CI | | [2.8-11.1] | [4.7-12.4] | [3.1-8.8] | [5.7-17.0] | [20.8-26.5] | [16.6-22.7] | [34.3-51.7] | [26.6-42.3] |
| Pre-obesity | | | | | | | | | |
| n | | 90 | 83 | 78 | 85 | 533 | 648 | 129 | 184 |
| $\hat{N}$ | | 69.874 | 62.199 | 89.365 | 111.194 | 1.054.117 | 1.455.824 | 340.180 | 323.414 |
| weighted % ^b^ | | 18.7% | 15.9% | 21.7% | 25.3% | 31.0% | 41.8% | 38.6% | 45.7% |
| 95% CI | | [13.7-23.8] | [11.4-20.4] | [16.3-27.1] | [18.7-31.8] | [27.9-34.1] | [38.1-45.5] | [30.7-46.6] | [38.5-53] |
| Underweight/normal weight | | | | | | | | | |
| n | | 615 | 602 | 240 | 217 | 786 | 537 | 63 | 79 |
| $\hat{N}$ | | 277.106 | 296.581 | 297.859 | 278.516 | 1.540.633 | 1.341.751 | 161.986 | 140.325 |
| weighted % ^b^ | | 74.3% | 75.6% | 72.4% | 63.3% | 45.3% | 38.5% | 18.4% | 19.8% |
| 95% CI | | [68.8-79.9] | [70.2-81.0] | [66.4-78.3] | [56.2-70.5] | [41.5-49.1] | [34.6-42.5] | [11.9-24.9] | [13.4-26.3] |
| p-value sex ^c^ | | p=0.534 | | p=0.069 | | p=0.051 | | p=0.213 | |

^a^ BMI categories defined according to the World Health Organization criteria.

^b^ Prevalence weighted for the distribution of the Portuguese population.

^c^ P-value comparing the prevalence of obesity vs. the other categories by sex.

n: sample size; $\hat{N}$: estimated population size; 95%CI: 95% confidence intervals
