# Supplementary material for: Prevalence of general and abdominal obesity in Portugal: comprehensive results from the National Food, nutrition and physical activity survey 2015–2016
Source: BMC Public Health. 2018 May 11;18:614. doi: 10.1186/s12889-018-5480-z (PMC5946450; doi:10.1186/s12889-018-5480-z)
Supplement: Supplementary file 2 — Figure S1. Prevalence of obesity observed and estimated by a regression model* from 3 months to 84 years. (DOCX 18 kb) [file 12889_2018_5480_MOESM2_ESM.docx]

Supplemental Figure 1. Prevalence of obesity observed and estimated by a regression model from 3 months to 84 years.

B-splines were fitted in a weighted logistic regression model. Knot points denote inflection of obesity prevalence.
